# The antioxidative enzyme SOD2 is important for physiological persistence of *corpora lutea* in lynxes

**DOI:** 10.1038/s41598-020-60634-x

**Published:** 2020-02-28

**Authors:** B. C. Braun, N. Halaski, J. Painer, E. Krause, K. Jewgenow

**Affiliations:** 1 Leibniz Institute for Zoo and Wildlife Research, Department of Reproduction Biology, Alfred-Kowalke-Str. 17, 10315 Berlin, Germany; 2 Leibniz Institute for Zoo and Wildlife Research, Department of Reproduction Management, Alfred-Kowalke-Str. 17, 10315 Berlin, Germany; 3 Present Address: Veterinary University Vienna, Research Institute for Wildlife Ecology, Savoyenstreet 1, 1160 Vienna, Austria; 4 Leibniz-Forschungsinstitut für Molekulare Pharmakologie, Robert-Rössle-Str. 10, 13125 Berlin, Germany

**Keywords:** Enzymes, Gene expression analysis, Immunohistochemistry

## Abstract

*Corpora lutea* (CL) are transient endocrine glands supporting pregnancy by progesterone production. They develop at the site of ovulation from the remaining follicle, are highly metabolically active and undergo distinct, transformative processes during their lifetime. In contrast to other species, CL of lynxes do not regress at the end of cycle, but remain functionally active (persist) for years. Reactive oxygen species (ROS) and anti-oxidative enzymes are described to be important for the functionality of CL. We examined ten anti-oxidative enzymes in fresh and persistent CL of lynxes as well as in domestic cat CL of different luteal stages. The gene expression profiles, especially those of SOD1 and SOD2, showed some remarkable differences between CL stages during non-pregnant and pregnant cycles of domestic cats and between fresh and persistent CL of lynxes. Lynx gene expression profiles of SODs were confirmed by western blot analysis, immunohistochemistry and activity assays. SOD2 was characterized by a conspicuous high expression and enzyme activity exclusively in persistent CL. We suggest that SOD2 is required to detoxify potential elevated superoxide anion levels by producing H_2_O_2_ in the physiologically persistent CL. This product might also act as a signaling molecule, securing the CL from apoptosis and insuring long-term luteal cell survival.

## Introduction

*Corpora lutea* (CL) are transient ovarian endocrine glands that are formed at the site of ovulation through transition of cells from the remaining disrupted follicle. By producing progesterone they support pregnancies^1^. These glands are highly metabolically active and undergo distinct, transformative processes^2^ during their lifetime of days (e.g. in rats), weeks (e.g. in dogs)^3^ or months (elephants)^4^. They pass different stages over formation, development/maintenance and regression; ending in the *corpus albicans* stage^2^, also described for domestic cat^5^. The latter stage usually confirms the end of a luteal phase. A very particular exception are lynxes, where persistent CL can be found^6–9^ that are not pathological as observed for other species like cows^10^. These persistent CL remain functionally active with continuous production of progesterone^11–13^. Possibly, this is part of a mechanism controlling the monooestrus in three of four lynx species^6^ and/or may be supporting early pregnancies of following cycles by their additional hormone production^14^. The persistence of these CL is confirmed to be several years^6^.

Reactive oxygen species (ROS) are a byproduct of steroid hormone production^15^ and anti-oxidative enzymes seem to protect the CL against luteolysis and apoptosis during the maintenance stage^16,17^. Furthermore it is described that ROS are elevated during luteal regression and that anti-oxidative enzymes can rescue CL from regression during pregnancy, see for review^16^. In cells, ROS like superoxide radical or hydrogen peroxide are a result of diverse activities. ROS are part in physiologically positive processes like signaling^18^ but they also show detrimental properties, particularly at non-physiological levels, hereby causing pathologies^19^. To keep the amount of ROS in balance, organisms use two categories of antioxidative defense components – enzymatic and non-enzymatic. The best known antioxidative enzymes are superoxide dismutases (SOD, type 1 and 2) which transform superoxide to hydrogen peroxide as well as catalase (CAT) and glutathione peroxidases (GPX) which reduce hydrogen peroxide to water^20^. Also other enzymes are involved in the conversion of hydrogen peroxide, like peroxidasin (PXDN)^21^. Additionally, other enzymatic reactions are described with contributions to the antioxidative system. Some of them regenerate oxidized versions of certain enzymes to their reduced form as thioredoxin (TXN). TXN is regenerated by thioredoxin reductases 2 (TXNRD2)^22^. Glutathione S-transferases (GST) transfer glutathione to different molecules. By such a reaction, glutathione S-transferase P (GSTP) detoxifies proteins after oxidative stress^23^ and is involved in the regeneration of peroxiredoxin 6 (PRDX6)^24^. Glutaredoxins are glutathione-dependent oxidoreductases^25^. The monothiol glutaredoxin 3 (GLRX3) has several functions, e.g. it is important for mouse embryo development and its deletion is embryonically lethal^26^.

ROS and therefore also anti-oxidative enzymes can play a role in the functionality of CL of mammalian species^27–31^. Sugino *et al*. noticed in his review that hydrogen peroxide or lipid peroxides inhibit progesterone production by luteal cells in rats and humans. Beside affecting enzymes of steroidogenesis, ROS could additionally cause direct harm to luteal cells by disrupting the plasma membrane, which is often seen in the regressing corpus luteum^32^. Nevertheless, species-specific differences are described. SOD1 was shown to be lowest in the regression phase of human CL, whereas the concentration of lipid peroxide was increasing^33^. In contrast, in mice enhanced lipid peroxidation was connected with an enhanced SOD activity during CL regression^28^. The aim of our study was to investigate, how the gene expression pattern of anti-oxidative enzymes changes during CL lifecycle of the domestic cat, when CL undergo a full functional and structural regression. Furthermore, we aimed to focus on the potential role of anti-oxidative enzymes in lynx CL persistence. Therefore, the expression of SODs, catalase and GPX4 was analyzed, since these enzymes were previously described to be important for CL function in other species^30,31,34^. Additionally, we studied the expression of other enzymes (PXDN, PRDX6, TXN, TXNRD2, GSTP and GLRX3) to get more insight in the anti-oxidative processes during domestic cat and lynx CL lifecycles.

## Results

### mRNA expression studies

All genes tested were expressed in cat (Table 1) and lynx (Table 2) luteal tissue.

**Table 1:** Quantitative real-time PCR analysis of antioxidative enzymes in different luteal stages of pregnant and non-pregnant cats.

*gene* sign. diff. pregn./non-pregn. stages	pregnancy			non-pregnant				
	pre-implantation	post-implantation	regression	formation	development/maintenance	early regression	late regression	*corpus albicans*
*SOD1 *-/*	279055.41 ± 142637.29	353137.78 ± 148327.65	164223.32 ± 156649.81	363202.99 ± 227155.24^A^	403090.67 ± 283576.68^A^	261443.57 ± 223994.98^A^	96264.42 ± 47396.00^B^	35935.51 ± 22007.44
SOD2 */*	1044.69 ± 208.78^a^	4031.86 ± 1821.65^b^	3376.87 ± 1929.21^b^	1116.99 ± 658.46^A^	2342.03 ± 1378.21^B^	3794.59 ± 3258.17^B^	1913.15 ± 796.43^A,B^	749.88 ± 297.59
CAT */-	2469.48 ± 749.74^a^	4611.96 ± 1513.09^b^	6022.58 ± 1540.29^b^	3619.59 ± 2390.10	3884.38 ± 1150.29	5165.31 ± 1755.71	5936.82 ± 2224.64	8026.04 ± 2031.86
*PXDN *-/-	196.16 ± 170.86	170.28 ± 81.95	161.44 ± 27.84	329.56 ± 399.25	159.11 ± 88.00	147.13 ± 85.51	162.56 ± 66.05	506.16 ± 243.18
*PRDX6 *-/-	1058.44 ± 293.12	1519.03 ± 471.96	1298.12 ± 458.34	1378.82 ± 585.61	1304.47 ± 333.42	1311.12 ± 482.96	1344.47 ± 472.71	1310.42 ± 483.15
*TXN* -/-	3631.43 ± 1184.65	3599.84 ± ± 1337.52	3405.07 ± 1735.11	4471.59 ± 3486.42	4764.10 ± 2635.44	3853.67 ± 2355.92	2593.69 ± 1059.94	4218.77 ± 2631.59
*TXNRD2 *-/-	276.19 ± 148.16	301.89 ± 126.10	250.45 ± 132.34	401.65 ± 326.48	323.69 ± 147.27	199.08 ± 90.21	216.78 ± 111.17	303.99 ± 114.29
*GPX4 *-/*	28757.67 ± 10456.08	17026.18 ± 6425.00	21765.76 ± 9833.01	34171.49 ± 18121.70^A^	22757.15 ± 10162.49^A^	21151.48 ± 8527.33^A^	12833.28 ± 4041.37^B^	6493.02 ± 1694.40
*GSTP* -/*	9430.30 ± 3624.31	10748.02 ± 3921.66	16568.57 ± 10051.22	14952.10 ± 10534.31^A,B^	8938.44 ± 3527.80^A^	18602.65 ± 7865.22^B^	18813.63 ± 8999.49^B^	13559.46 ± 5254.88
*GLRX3 *-/-	128.63 ± 93.19	174.68 ± 60.03	221.59 ± 57.58	197.57 ± 146.45	151.20 ± 59.17	243.88 ± 113.66	286.66 ± 122.86	460.99 ± 162.88

Mean values ± standard deviations per luteal stage are depicted as relative mRNA levels referring to 1 ng original total RNA. *: statistical differences (p <0.05) between luteal stages of pregnancy respectively non-pregnancy, -: no statistical differences between luteal stages. Different superscripts (a, b for pregnant stages, A, B for non-pregnant stages) represent significantly different values between the groups (p <0.05). *Corpus albicans* stage could not be related to pregnancy or non-pregnancy and was therefore excluded from statistical analysis. *SOD1*: superoxide dismutase 1, *SOD2*: superoxide dismutase 2, *CAT*: catalase, *PXDN*: peroxidasin, *PRDX6*: peroxiredoxin 6, *TXN*: thioredoxin, *TXNRD2*: thioredoxin reductase 2, *GPX4*: glutathione peroxidase 4, *GSTP*: glutathione S-transferase P, *GLRX3*: glutaredoxin 3.

**Table 2:** Quantitative real-time PCR analysis of antioxidative enzymes in different luteal stages of lynx.

*gene* sign. diff. IL1/IL2/EL1	IL1 fresh, f	IL1 persistent, d/m	IL2 fresh, f	IL2 persistent, d/m	EL1 fresh, d/m	EL1 persistent, d/m + er	ELG persistent, er
*SOD1* */*/*	173400.58 ± 39215.24	270425.75 ± 68107.80	171776.86 ± 46803.35	405171.57 ± 51034.03	996101.10 ± 332123.69	405762.07 ± 292431.47	497872.30 ± 247253.31
SOD2 */*/-	2860.29 ± 489.20	33360.97 ± 14471.34	4379.36 ± 863.56	80531.32 ± 20620.20	5822.41 ± 1752.86	7438.06 ± 5068.92	18613.40 ± 11323.73
*CAT *-/-/-	3142.37 ± 124.86	3986.28 ± 930.71	3296.98 ± 745.29	3055.16 ± 744.59	7034.37 ± 2366.71	15672.90 ± 13578.53	4126.20 ± 1825.65
PXDN */*/-	137.58 ± 11.56	78.31 ± 18.14	200.37 ± 56.79	65.89 ± 24.25	194.71 ± 67.64	228.01 ± 107.39	131.63 ± 35.14
PRDX6 */*/-	1490.51 ± 110.58	2338.54 ± 351.62	1067.31 ± 209.05	2394.01 ± 535.54	2390.07 ± 351.36	3385.26 ± 761.49	2070.52 ± 742.62
*TXN* -/-/-	1699.38 ± 277.10	1673.57 ± 540.24	2328.20 ± 1305.09	1056.94 ± 460.73	20563.82 ± 2189.20	18513.49 ± 3543.42	4076.39 ± 2557.62
TXNRD2 */-/-	133.20 ± 19.72	236.00 ± 59.65	147.33 ± 39.52	167.78 ± 56.40	277.43 ± 24.39	212.80 ± 75.58	146.70 ± 104.49
*GPX4* -/*/-	32513.17 ± 5960.22	37223.86 ± 7224.04	29220.62 ± 9845.40	48028.83 ± 7164.17	72393.89 ± 21397.31	46731.25 ± 20932.81	62904.10 ± 20667.91
GSTP */*/-	3443.67 ± 381.04	15763.73 ± 3174.02	4082.85 ± 764.28	13725.38 ± 4791.09	23684.31 ± 6871.83	36227.95 ± 24618.03	26845.17 ± 14412.08
*GLRX3* */-/*	93.86 ± 17.04	148.39 ± 36.31	150.18 ± 69.40	99.48 ± 40.50	404.04 ± 35.18	695.01 ± 196.20	210.72 ± 115.90

Mean values ± standard deviations per luteal stage are depicted as relative mRNA levels referring to 1 ng original total RNA. *: statistical differences (p <0.05) between fresh and persistent CLs of one animal, -: no statistical differences between luteal stages. IL: Iberian lynx in breeding season (1: animal 1, 2: animal 2), EL: Eurasian lynx (1: animal 1 of breeding season, G: grouped samples of 5 animals outside of breeding season), f: formation, d/m: development maintenance, er: early regression. *SOD1*: superoxide dismutase 1, *SOD2*: superoxide dismutase 2, *CAT*: catalase, *PXDN*: peroxidasin, *PRDX6*: peroxiredoxin 6, *TXN*: thioredoxin, *TXNRD2*: thioredoxin reductase 2, *GPX4*: glutathione peroxidase 4, *GSTP*: glutathione S-transferase P, *GLRX3*: glutaredoxin 3.

**Figure 1:**
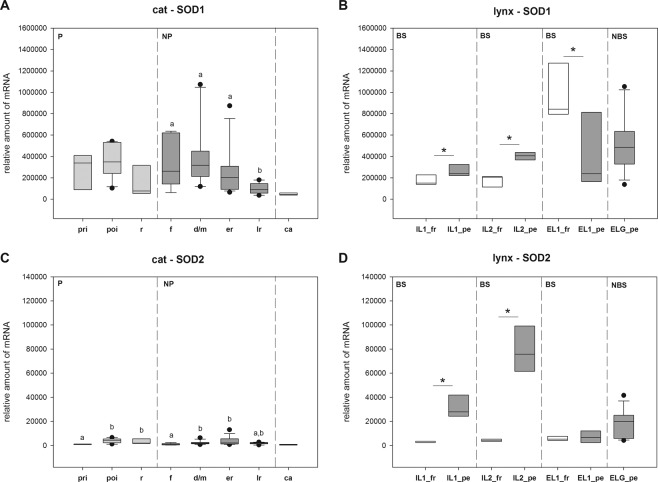
Intraluteal mRNA expression of *SOD1* and *SOD2* in domestic cat and lynx samples of different CL stages. Mean values (±SD) refer to 1 ng original total RNA. P-values are calculated from the Kruskal-Wallis rank sum test, significant differences between stages are calculated from post hoc pairwise comparisons (P-value adjustment: Benjamini-Hochberg) for cat samples and with Mann-Whitney U test for lynx samples. Bars marked by different superscripts (**A**,**B**,*) are significantly different (p <0.05). P: pregnancy, NP: non-pregnant luteal phase, BS: breading season, NBS: non-breeding season, pri: pre-implantation period, poi: post-implantation period, r: regression, f: formation, d/m: development/maintenance, er: early regression, lr: late regression, ca: *corpus albicans*. IL: samples of Iberian lynxes 1 and 2, EL1: samples of Eurasian lynx 1, ELG: samples of Eurasian lynx group, fr: fresh CL, pe: persistent CL.

In the cat, statistically different expressions were found for CL-stages of pregnancy for *CAT* and for stages of the non-pregnant cycle for *SOD1*, *GPX4* and *GSTP4*; (see Table 1, *SOD1*: see also Fig. 1A). Only one gene (*SOD2*) was differentially expressed between stages of both, pregnant and non-pregnant cycles (Table 1, see also Fig. 1C). *SOD2* expression was lower in the formation (non-pregnant cycle) and preimplantation (pregnancy) stages compared to the later phases — the development/maintenance and early regression stages in non-pregnant-cycle and the post-implantation and regression stages during pregnancy. In the late regression stage of non-pregnant cycle it was decreasing again (Table 1 + Fig. 1C). Studying gene expression in samples of *corpora albicantia* revealed that some genes were nearly equally expressed compared to all stages in pregnant and non-pregnant cycles (*PRDX6*, *TXN*, *TXNRD2*, *GSTP*). *SOD1*, *SOD2* and *GPX4* were noticeable less expressed, and expression *of CAT*, *PXDN* and *GLRX3* was increased (Table 1, *SOD1* and *SOD2*: see also Fig. 1A,C).

In lynxes, differences between fresh and persistent CL were analyzed per animal (EL1, IL1, IL2). To assess the results correctly it must be taken into account that the fresh CL of Iberian lynx samples represent a different lifecycle stage (IL1, IL2: formation stage) compared to the fresh CL of Eurasian lynx sample (EL1: development/maintenance stage) and also the persistent CL stages slightly differ (IL1, IL2: development/maintenance stage, EL1: development/maintenance and/or early regression stage). Although we observed differences for *SOD1* for all three comparisons within individuals (Table 2, see also Fig. 1B), the expression in fresh CL was lower in IL1 and IL2 but higher in EL1 compared to corresponding persistent CL. For *PXDN*, the expression in fresh CL samples of Iberian lynx was higher than in the persistent CL; the opposite was observed for *SOD2*, *PRDX6* and *GSTP* (Table 2, *SOD2* see also Fig. 1D). For *TXNRD2*, *GPX4* and *GLRX3* only samples of one IL showed expression differences (Table 2). In EL1, *GLRX3* was lower expressed in fresh CL compared to persistent ones (Table 2). Values of EL samples from non-breeding season (ELG) were usually in the range of the persistent CL of IL1, IL2 and EL1 (Table 2, *SOD1* and *SOD2*: see also Fig. 1B,D).

### Western Blot analysis of SOD1 and 2, in-gel SOD2 activity assay, total SOD activity

**Figure 2:**
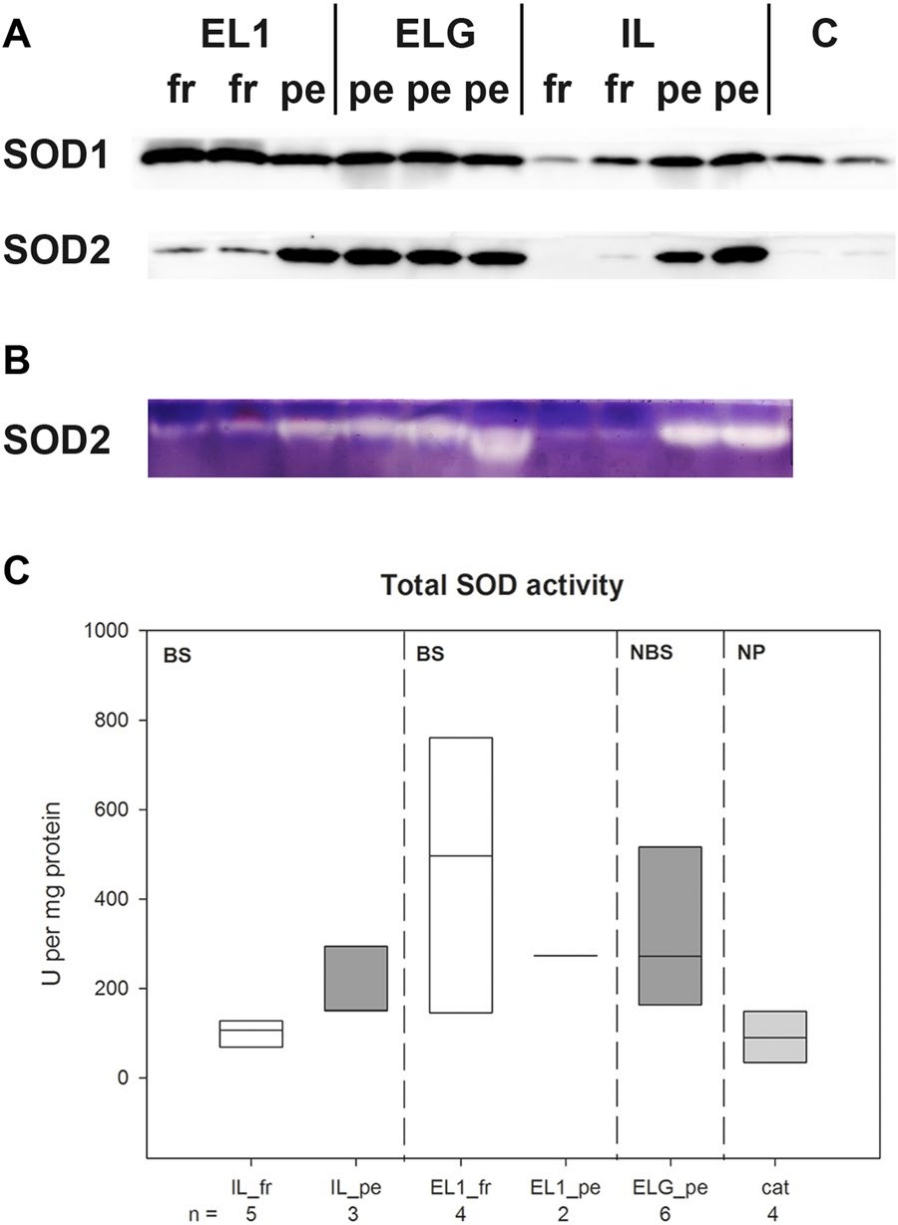
Western Blot (**A**) for SOD1 and SOD2 detection and in-gel activity assay (**B**) for SOD2 activity detection with CL homogenate samples of lynxes. Per lane 10 µg (Western Blot) or 20 µg (in-gel activity assay) protein were applied. (**C**) Enzymatic assay for total SOD. EL1: Eurasian lynx 1, ELG: samples of Eurasian lynx group, IL: samples of Iberian lynxes, C: samples of domestic cat CL. fr: fresh CL, pe: persistent CL, BS: breading season, NBS: non-breeding season, NP: non-pregnant cycle.

Western blot for SOD1 protein detection revealed only slight differences between stages. The SOD1 content in fresh CL of Iberian lynx seemed to be slightly lower than in the persistent CL whereas the protein expression in fresh CL of EL1 was somewhat higher compared the corresponding persistent CL (Fig. 2A, Sup. Fig. 1).

Differences between fresh and persistent CL were more pronounced for SOD2 protein expression. Both, for Iberian and Eurasian lynx fresh CL, SOD2 western blot signals (Fig. 2A) were weak whereas the signals of persistent CL were very strong. This pattern was mirrored by the in-gel activity assay for SOD2 (Fig. 2B, Sup. Fig. 2). Total SOD activity was highest in fresh CL samples of EL1 (development/maintenance stage) and lowest in fresh CL of Iberian lynxes (formation stage) (Fig. 2C). Values of persistent CL were determined to be within this range.

### Immunohistochemistry

**Figure 3:**
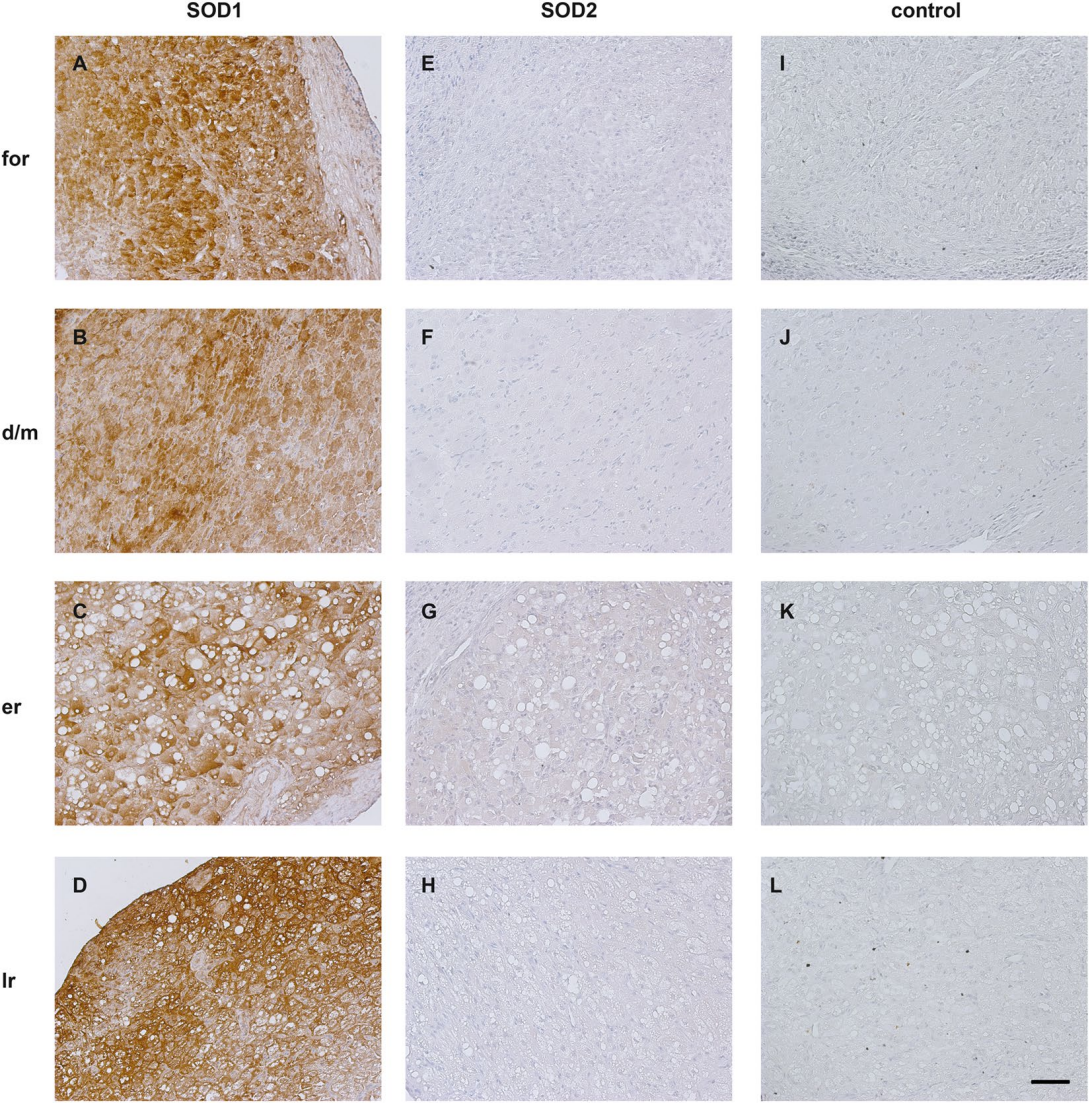
Immunohistochemical localization of superoxide dismutases 1 (SOD1) and 2 (SOD2) in different stages of domestic cat CL. f: formation; d/m: development/maintenance; er: early regression; lr: late regression; Bar: 50 µm, valid for all images.

**Figure 4:**
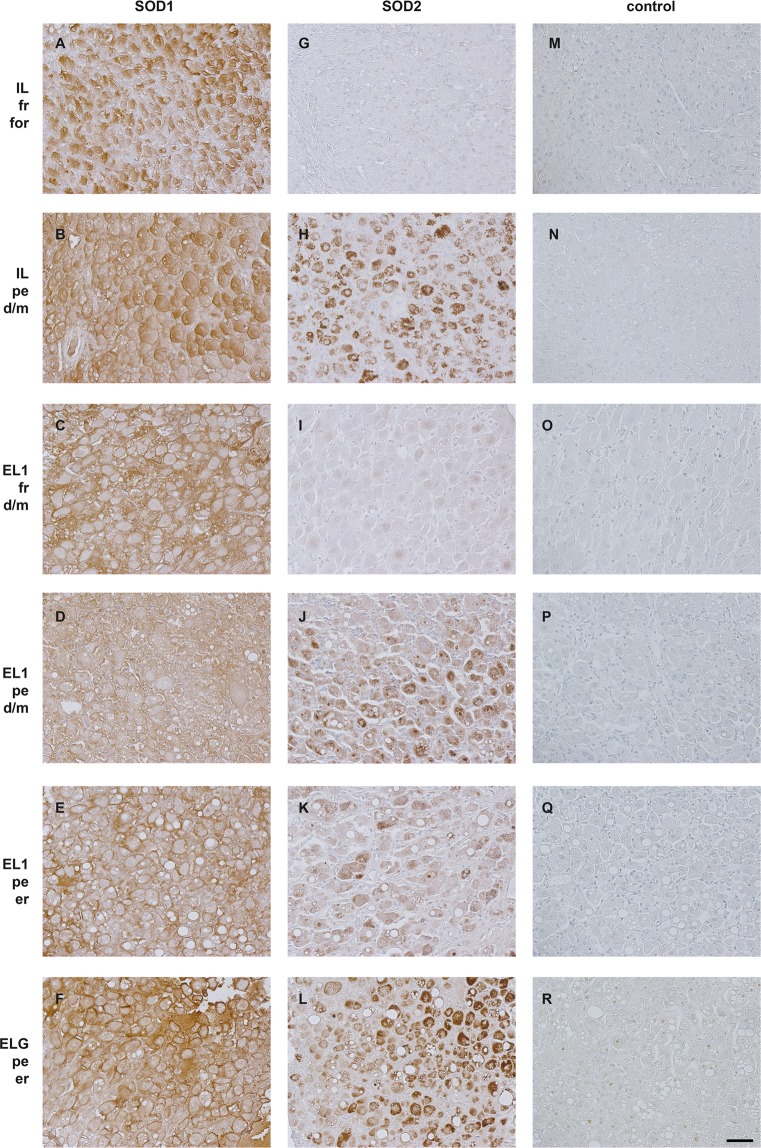
Immunohistochemical localization of superoxide dismutases 1 (SOD1) and 2 (SOD2) in lynx CL. IL: samples of Iberian lynxes, EL1: samples of Eurasian lynx 1, ELG: samples of Eurasian lynx group, fr: fresh CL, pe: persistent CL, for: formation, d/m: development/maintenance, er: early regression. Bar: 50 µm, valid for all images.

Immunohistochemistry revealed different staining patterns for lynx and cat samples. SOD1-signals were present in all cat CL samples but the staining intensity was not only slightly different between samples but also depending on the regions of CL. (see Fig. 3A–D). The staining of SOD1 on lynx samples was diverse too (Fig. 4A–F). Interestingly, for a fraction of cells the staining was more pronounced intracellular (Figs. 3A–C and 4A,B), while for the others the cell membrane was more accentuated (Fig. 4C–F).

The SOD2 staining was almost non-detectable on cat samples. Only during early regression (Fig. 3G) a slight staining was visible; at other stages no signal was detected (Fig. 3E,F,H). In contrast to cats, SOD2 was detectable on luteal cells of most lynx samples and showed clear differences between stages. On freshly formed CL in formation stage (IL, Fig. 4G) almost no SOD2-staining was visible, whereas luteal cells in the development/maintenance stage of fresh CL (EL1) were stained with variable intensities (Fig. 4I). In contrast, a very strong staining was observed on persistent CL of lynxes (Fig. 4H,J–L). On control sections (no primary antibodies), no specific signals were detected (Figs. 3I–L and 4M–R).

### Protein identification by mass spectrometry

All enzymes that were analyzed on gene expression level could be uniquely identified by tandem mass spectrometry. In Table 3 the detected numbers of tryptic peptides per protein are listed. Furthermore, relative quantification for different pairs of samples was achieved using the LFQ algorithm and the resulting ratio values are shown in Table 3. A statistical analysis was not possible due to the low n-number but some tendencies can be described. SOD2 and GSTP seemed to be obviously higher expressed in persistent CL compared to fresh CL, in both, Iberian and Eurasian lynx CL samples. For TXN a clearly higher expression in persistent CL could be shown for three of four tested pairs. Although peptides and normal intensities were detected for PXDN and GLRX3 in persistent CL of EL1, the LFQ intensities were defined as zero. The same was true for GPX4 in fresh EL1 samples. GLRX3 and PXDN showed a trend for higher expression in fresh CL. The LFQ-ratio values differed substantially between analyzed Iberian and Eurasian lynx CL pairs for TXNRD2. For some proteins (SOD1, CAT, PRDX6) no clear differences between persistent and fresh CL could be detected. In Supplemental Table 1 additional data to illustrate the MS analysis of these 10 proteins is provided.

**Table 3:** Mass spectrometry analysis of lynx CL. For proteins analyzed in this study, numbers of peptide counts as well as the ratio of Label-free quantification values (LQF) per CL pair are listed. The values of one persistent (pe) versus one fresh (fr) CL of each Iberian lynx (IL1 and IL2) and of each ovary (-1, -2) of the same Eurasian lynx (EL1) were compared.

Gene name	Protein IDs suggested by MaxQuant analysis	Peptide counts (unique)	LQF-ratios			
			IL1 pe/fr	IL2 pe/fr	EL1-1 pe/fr	EL1-2 pe/fr
*SOD1*	XP_006935984.1, PREDICTED: superoxide dismutase [Cu-Zn] [*Felis catus*]	12	1,20	0,55	0,54	1,97
*SOD2*	XP_019687004.1, PREDICTED: superoxide dismutase [Mn],mitochondrial [*Felis catus*]	19	4,93	6,18	3,31	54,22
*CAT*	XP_003993206.1, PREDICTED: catalase [*Felis catus*]	25	1,07	0,71	0,63	2,18
*PXDN*	XP_019683554.1, XP_006930569.1, PREDICTED: peroxidasin homolog isoforms X1 and X2 [*Felis catus*]	9;9	0,91	0,19	*	*
*PRDX6*	XP_011289183.1, PREDICTED: peroxiredoxin-6 [*Felis catus*]	25	1,25	1,71	1,08	2,18
*TXN*	XP_011286804.1, PREDICTED: thioredoxin [*Felis catus*]	7	4,75	1,23	5,89	2,04
*TXNRD2*	XP_006938766.1, XP_019670460.1, XP_019670459.1, XP_019670458.1, XP_019670457.1, XP_019670456, XP_006938765.1, PREDICTED: thioredoxin reductase 2, mitochondrial isoforms X1 - X6 [*Felis catus*]	14;14;14;14;14;14;14	2,57	2,94	1,31	0,64
*GPX4*	XP_011286531.2, PREDICTED: phospholipid hydroperoxide glutathione peroxidase, mitochondrial, partial [*Felis catus*]	10	5,58	0,30	#	#
*GSTP*	XP_011285433.1, XP_019668324.1, PREDICTED: glutathione S-transferase P [*Felis catus*]	11;11	3,68	3,59	2,17	33,77
*GLRX3*	XP_019669563.1, PREDICTED: glutaredoxin-3, partial [*Felis catus*]	7	0,53	0,77	*	*

^*^LQF values of persistent CL was zero, #: LQF-values of fresh CL was zero.

## Discussion

We were able to confirm the expression of all tested anti-oxidative enzymes in cat and lynx CL. To our knowledge, this is the first comprehensive analysis of a substantial number of different anti-oxidative enzymes with the aim to elucidate their role in CL. CL are endocrine glands which are characterized by a very dynamic life cycle characterized by different stages that alter - depending of the species - in relatively short time periods. Because of the latter, the anti-oxidative system is considered to be important to ensure tissue homeostasis in these highly active metabolic glands.

Unexpectedly, only some of the tested enzymes showed stage-dependent profiles, indicating that not all components of the anti-oxidative system - although likely being involved in the CL function - are influenced in their expression through conditions caused by an altered CL stage. Overall, most interesting results were observed for SODs, consequently leading to a more detailed examination on protein level for these enzymes.

The importance of SOD within CL of different species was already described in literature, including its differential expression between species and between CL of pregnancy and of non-pregnant/pseudopregnant cycle^16,27,29^. Also the expression/activity profiles for SOD1 and SOD2 were often different^27,30,33^; maybe due to differences in their regulation. A number of (potential) transcription factors are listed in the review of Miao & St. Clair^35^, some of them are regulators for both SOD types. NF-κB, for instance, is likely one of the most important transcriptional factors regulating SOD2 induction^36^, but can also play a role for SOD1 expression^37^. Other factors seem to be more unique, like arachidonic acid or the proliferator-activated receptor γ (PPARγ)^35^. Arachidonic acid binds to the peroxisome proliferator-responsive element (PPRE) in 5′-flanking sequence of SOD1 gene^38^, whereas PPARγ is described to be relevant for SOD2 expression^39^. Preliminary data (Supplemental Fig. 3) showed a marked difference between fresh and persistent CL regarding NF-κB2 protein expression, although the meaning of its partly different cellular localization as well as its function for SOD1 or SOD2 expression in luteal cells is still unclear.

In human CL, SOD1 activity was highest in mid cycle and lowest during regression^33^. In contrast to this, SOD2 activity in human CL increased towards late luteal phase and regression^33^. In the rat, SOD1 activity showed a peak around day 12 of pregnancy and day 9 in pseudopregnancy, decreasing afterwards. SOD2 reached highest level on day 12 in pregnancy too but stayed thereafter more or less constant, whereas in pseudopregnancy it was decreasing towards regression after reaching highest values on day 11^27^. It was suggested that the decrease in SOD1 and the increase in ROS are involved in functional luteolysis of rat CL^27^. Al-Gubory *et al*. studied ovine CL. The activity of both, SOD1 and SOD2 increased over normal cycle^29^, but during the analyzed pregnancy period of 128 days, the SOD1 reached a plateau from day 40 onwards and for the SOD2 activity no significant changes were detected in the studied period^16^. Measurement of total bovine SOD activity revealed a peak on day 16^40^, this seemed to match more with SOD1 protein expression, which was highest in mid and late cycle (day 8–17) compared to SOD2 expression which increased over estrus cycle^30^. The bovine mRNA expression SOD profiles differed a little bit to the corresponding protein expression. Both mRNA profiles were similar, with highest expression in the mid-phase of estrus cycle^30^.

For cat CL samples we did not determine total SOD or SOD2 activity because the lack of enough sample material. However, the SOD2 gene expression profile of non-pregnant luteal cycle (Fig. 1C) seemed to be comparable to that of bovine estrus cycle whereas this is less true for the bovine SOD1 profile^30^ which slightly deviated to our results (Fig. 1A). In the cat, we found diverging profiles for *SOD1* and *SOD2*, as well as for both, pregnant and non-pregnant luteal cycles.

In lynxes, immunohistochemical signals for SOD1 did not differ markedly in intensity, but a heterogeneous localization of signals was visible, since both cytoplasmic and extracellular staining was detected. Samples of IL rather showed a more pronounced intracellular localization along with extracellular expression whereas for Eurasian lynx the extracellular staining was predominant. Nevertheless, despite the description of SOD1 being a cytoplasmic enzyme, there are studies, summarized in the review of Mondola *et al*.^41^ which described its extracellular secretion. Extracellular SOD1 can activate through its interaction with a receptor cellular pathways^41^. Potentially, our staining pattern reveals a similar mechanism for feline CL, but this has to be confirmed by future examinations. The western blot signals of SOD1 as well as the total SOD activity were in accordance to gene expression profile of SOD1 of lynx samples. Fresh CL of formation stage (IL) had the lowest signals, and fresh CL of development/maintenance (EL1) the highest, all persistent CL showed an expression in between. If SOD1 would play an important role for persistency, a more pronounced expression in persistent CL would be expected. In many species, SOD1 profiles were often related to the progesterone profile, thus to the metabolic activity of luteal cells. In rat luteal cells, SOD1 expression was correlated with progesterone production^42^. In addition, the serum progesterone profile during pregnancy was parallel to SOD1 activity in rat CL^27^. For cyclic bovine CL, SOD and catalase activities showed patterns most similar to plasma progesterone^40^. A strong relation of SOD1 expression to progesterone production, however, seems to be unlikely in lynx CL. In contrast to SOD1 expression, intraluteal progesterone content of freshly formed CLs in Iberian lynxes was higher than that of the corresponding persistent CL^43^. Likewise, in the domestic cat, gene expression profile of SOD1 did not completely fit to the intraluteal progesterone content with highest values detected in development/maintenance stage^44^.

In contrast to SOD1, the differential expression of SOD2 hints to its specific role for persistency of CL in lynxes. Immunohistology of SOD2 revealed the strongest protein expression in persistent CL, accompanied by highest SOD2 in-gel activity and also higher gene expression (the latter at least in the Iberian lynx). In particular, the formation stage is characterized by very low protein expression.

The question remains, why SOD2 is relatively highly expressed and active in persistent CL? SOD2 is the mitochondrial SOD, located in the matrix^45,46^. Also the dotted staining pattern of SOD2 signals in our immunohistochemistry hinted to mitochondrial localization. In mitochondria, high amounts of superoxide anion - a substrate of SODs - originate from processes of the mitochondrial electron transport system^46^. Superoxide anion is converted by SOD to H_2_O_2_. A higher SOD2 activity could indicate the requirement to deal with elevated amounts of superoxide anions. Its potential source - the mitochondrial electron transport system - is responsible for ATP generation^47^. Whether indeed - and if so, why - persistent CL produce more ATP is so far unknown. In addition, in steroidogenic cells, mitochondrial P450 enzyme activities in connection with adrenodoxin reductase and adrenodoxin contribute to free radical generation and thereby to superoxide anion formation in a substantial manner^15^. According to Hanukoglu *et al*.^15^, the relevant P450 enzyme for producing superoxide anion in luteal cells is CYP11A1 (Cholesterol side chain cleavage enzyme). We demonstrated that the gene expression of CYP11A1 was significantly elevated in persistent CL samples of Iberian lynx compared to the fresh CL, although the expression in Eurasian lynx samples was less pronounced^12^. In contrast, immunohistochemistry for CYP11A1 did not show stronger signals in persistent CL of both lynx species compared to fresh Iberian lynx CL samples^12^. We would also deny a role of other P450 enzymes involved in sexual steroid synthesis, like CYP17A1 and CYP19A1 as their expression profiles^12^ also did not follow the SOD2 expression profile observed in the present study. Therefore, we suggest that the high SOD2 activity is not necessarily coupled to CYP11A1 or other steroidogenic enzyme activity, but is rather linked to ATP production in persistent CL of lynxes.

It has been shown that ROS express positive physiological roles, acting partly as signaling molecules or anti-microbiotics. At high concentrations, however, they lead to pathological responses and cell death, as summarized for H_2_O_2_^48^. Both actions were designated as oxidative eustress and distress by Sies^49^. Maybe the H_2_O_2_ produced by SOD fulfills in CL, besides being a metabolite from superoxide anion only, signaling functions and is therefore CL-protective. As the total SOD activity does not fit to the SOD2 in-gel activity profile, SOD2 may not influence total superoxide dismutase activity and accordingly total intracellular H_2_O_2_ level. But the slight changes in H_2_O_2_ concentration caused by SOD2 activity could be sufficient enough to ensure effects, at least locally in the mitochondria. H_2_O_2_ can cross membranes freely and the effects of H_2_O_2_ and superoxide anion produced in mitochondria are described in the reviews of Zou *et al*.^46^ and Reczek & Chandel^50^; e.g. H_2_O_2_ can oxidize critical cysteine thiol groups of phosphatases^50^. Moreover, Zwacka *et al*. showed that the overexpression of SOD1 and SOD2 in human lung epithelial cell line reduced the level of apoptosis post-irradiation compared to control cells^51^. Furthermore, SOD2 provided protection against TNF-induced cytotoxicity and some but not all kinds of apoptosis as summarized in a review of Sinha *et al*.^52^. In conclusion, the elevated SOD2 activity in persistent CL could potentially prevent apoptosis of luteal cells and could hereby promote persistence. This is in line with our former study in which a higher gene expression of the pro-survival factor B-cell CLL/lymphoma 2 (BCL2) in persistent CL of lynxes compared to fresh CL^43^ was reported.

In summary, we suggest a specific role for SOD2 in persistent CL of lynxes. Whether it deals with the “classical” anti-oxidative enzyme function to detoxify a potential elevated ROS level in these CL, or the generated H_2_O_2_ has regulatory and thus luteotrophic functions, has to be further elucidated in future studies.

## Material and methods

### Tissue collection and determination of developmental stage

Ovaries of domestic cats (mainly stray cats) were obtained from local animal shelters and clinics after ovariectomy or ovariohysterectomy for the purpose of permanent contraception. These treatments were not related to the purpose of the experiment. Castrations are compliant with the “Protection of Animals Act” in Germany; no further guidelines had to be considered. Transport, sample preparation and staging were described before^5^. Per animal only one CL was used, samples are listed in Supplemental Table 2.

Collection of Iberian lynx (*Lynx pardinus*) CL samples in Spain (two animals, IL1 and IL2, 7 days after mating) and of Eurasian lynx (*Lynx lynx*) samples (CL of 5 animals, before mating season) in Norway (ELG = Eurasian lynx group) is described in Zschockelt *et al*.^12^.

Additional to these previously described lynx samples, CL of another Eurasian lynx (EL1) were used in the present study. This lynx died due to illegal hunting in Germany and was brought to the pathology of the Leibniz-Institute of Zoo and Wildlife Research for examinations. This animal was recognized as pregnant. The gestation chambers had an outer diameter of around 15 mm and crown-rump-length of the fetuses was about 4 mm. In the domestic cat these values correspond to pregnancy day 17/18^53^. Although pregnancy length is as long as in the domestic cat, lynx cubs are bigger at parturition^54,55^. Thus, it might be assumed that embryo growth in lynx is quicker compared to the cat and therefore we set the time frame for these 4 mm embryos to d15–d18 of pregnancy. From both ovaries of this animal fresh and persistent CLs were isolated. Histology revealed that the fresh CL were in development/maintenance stage, the persistent CL were classified as development/maintenance and/or early regression stage. The summary of lynx samples is listed in Supplemental Table 2.

### Sequence analysis

Total RNA isolation from CL tissue and reverse transcription to cDNA was performed as previously described^44^. Primers (Table 4) were designed based on predicted feline genes sequences listed in GenBank. Based on feline single strand cDNA templates, partial cat and lynx cDNA sequences were amplified using the Expand High FidelityPLUS PCR system (Roche Diagnostics Deutschland GmbH, Mannheim, Germany) as described before^56^ or DreamTaq Hot Start DNA Polymerase (Thermo Fisher Scientific, Darmstadt, Germany). The PCR conditions for the Expand High FidelityPLUS PCR system were 94 °C for 2 min followed by 35 cycles of denaturation at 94 °C for 60 s, 45 s or 20 s (gene dependent), annealing for 60 s, 45 s or 20 s (temperatures are listed in Table 4), elongation at 72 °C for 120 s, 100 s, 90 s or 45 s and a final elongation at 72 °C for 7 min. Using DreamTaq the conditions were 2 min denaturation at 95 °C followed by 35 cycles with denaturation at 94 °C for 30 s, 30 s at 60 °C annealing and 60 s at 72 °C elongation followed by a final 10 min elongation step at 72 °C. Purified PCR products of most genes were ligated to the pCR2.1-TOPO TA vector and transfected into one shot TOPO10 cells (both Thermo Fisher Scientific) or DH5 alpha cells (Thermo Fisher Scientific). PCR product of *Lynx pardinus CAT* was ligated to the pJet 1.2 vector (Thermo Fisher Scientific), followed by transformation into DH5 alpha cells. Selected positive clones were sequenced by the Services in Molecular Biology GmbH (Dr. M. Meixner, Rüdersdorf, Germany). All other PCR products were sequenced, but not cloned. Sequence information of genes were submitted to GenBank, the corresponding IDs are listed in Table 4.

**Table 4:** Sequences of PCR primers.

Gene	GenBank ID	Species	Primer sequence 5′ – 3′	T_A_ (°C)	Product size (bp)	Use
*SOD1*	MH882489^#^ MK574053 MK574054	*Felis catus* *Lynx lynx* *Lynx pardinus*	SOD1-fw: GAG CAT GGA TTC CAC GTC C SOD1-rv: CTC AGA TCG CAT CCT AGG G SOD1-q-fw: GAG AGG CAT GTT GGA GAC CT SOD1-q-rv: GTC ATC TCG TTT CTC GTG GAC	53 59.5	363 144	a b
*SOD2*	MK574050 MK574051 MK574052	*Felis catus* *Lynx lynx* *Lynx pardinus*	SOD2-fw: GGC AGA AGC ACA GCC TCC SOD2-rv: TTC TGC TCA GTG TAA TGA TGT SOD2-q-fw: CAC ATC AAC GCC CAG ATC SOD2-q-rv: CAC CCT TAG GGC TCA GGT T	53 58.5	624 208	a b
*CAT*	MK574073 MK574074 MK574075	*Felis catus* *Lynx lynx* *Lynx pardinus*	CAT-fw: CCA GCA ACG TTC TGC GAA G CAT-rv: CTG CTT CAC AGG TGG AGA G CAT-q-fw: CTG AAG GAT CCG GAC ATG CAT-q-rv: GTG TCC ATC TGG AAT CCC T	55/53 56.5	1604 102	a b
*PXDN*	MK574055 MK574056 MK574057	*Felis catus* *Lynx lynx* *Lynx pardinus*	PXDN-fw: AAG GGA CTT GCC TCT CTA GA PXDN-rv: GTC ACC TGA ACC CCA TCC T PXDN-q-fw: CGA GCT GAG CAT GAA CAC A PXDN-q-rv: CCA GCA CCT CCG TGT TCT	60 59.5	1307 212	a b
*PRDX6*	MK574058 MK574059 MK574060	*Felis catus* *Lynx lynx* *Lynx pardinus*	PRDX6-fw: CCA ACT TCG AGG CCA ATA CT PRDX6-rv: GCA GGA GAA CAT GAC TGG C PRDX6-q-fw: GAA GAC CAT CTT GCC TGG A PRDX6-q-rv: GGT CCA GCA TGC CTA ACA G	53 59	706 132	a b
*TXN*	MK574047 MK574048 MK574049	*Felis catus* *Lynx lynx* *Lynx pardinus*	TXN-fw: GTA TGC TTT TCA GGA AGC CTT TXN-rv: GGC TGG TTA TGT TTT CAG AAA A TXN-q-fw: GTG GTG TGG ACC TTG CAA A TXN-q-rv: GGC ATG CAT TTG ACT TCA CA	60 60.5	322 135	a b
*TXNRD2*	MK574044 MK574045 MK574046	*Felis catus* *Lynx lynx* *Lynx pardinus*	TXNRD2-fw: GCT GCA TCC CCA AGA AGC T TXNRD2-rv: CGA CCT ATG GCC CAC AGG TXNRD2-q-fw: CAG CTT TGT CAA TGA GCA CAC TXNRD2-q-rv: CCT TCA GCC AGA AGA TGT CAT	60 59	685 169	a b
*GPX4*	MH882486^#^ MK584627 MK584628	*Felis catus* *Lynx lynx* *Lynx pardinus*	GPX4-fw: CTG TGC TCA GTC CAT GCA C GPX4-rv: CTT GTG GAG CTA GAG GTA G GPX4-q-fw: CTT GCA ACC AGT TCG GGA G GPX4-q-rv: CTT GGG CTG GAC TTT CAT CC	53 58.5	496 154	a b
*GSTP*	MK574067 MK574068 MK574069	*Felis catus* *Lynx lynx* *Lynx pardinus*	GSTP-fw: GAG GCC ATG CGC ATG CTG GSTP-rv: CTG AA ACT CTC ACT GCT TC GSTP-q-fw: GGC TAT ACG GGA AGG ACC A GSTP-q-rv: CAG CAG CGT CTC GAA AGG	53 59.5	597 170	a b
*GLRX3*	MK574070 MK574071 MK574072	*Felis catus* *Lynx lynx* *Lynx pardinus*	GLRX3-fw: TTG TGA AGT TGG AAG CTG AAG GLRX3-rv: CCC TTT CAC ATA SAG CTG AG GLRX3-q-fw: GTT CAG CGA CAC GCA TCT AG GLRX3-q-rv: GGA TTT CCA CCA TCT GCT TG	60 59.5	734 169	a b

fw: forward; rv: reverse, TA: annealing temperature.

^a^Used for sequence analysis.

^b^Used for expression studies.

^#^analyzed in Hryciuk *et al*.^66^.

### Quantitative real-time PCR

Homogenization, followed by total RNA extraction and reverse transcription to cDNA for ELG and IL samples was already described in previous studies^5,12,44^. Samples of EL1 were handled identically. No-reverse transcription controls were included to test for genomic DNA contamination. Intron-spanning primers for quantitative real-time PCR (qPCR) were designed according to sequences identified in the present study (Table 4). For qPCR, diluted cDNA (according to 1 ng of total RNA) was analyzed with the CFX96 Real-Time PCR Detection System using the SsoFast EvaGreen Supermix (both from Bio-Rad Laboratories GmbH, Munich, Germany; for detailed description see^57^). The qPCR conditions were: 98 °C for 2 min and 40 cycles of 8 s at 98 °C and 8 s at different annealing temperatures (Table 4). Quantification of qPCR products was performed using the CFX Manager Software 1.6 (Bio-Rad Laboratories GmbH). Serial dilutions of plasmid DNA carrying genes of interest sequences or of PCR products were used for calibration.

Glyceraldehyde 3-phosphate dehydrogenase (*GAPDH*), Glutaminase (*GLS*), TATA box binding protein (*TBP*), β-actin (*BACT*), and ribosomal protein S7 (*RPS7*) were tested as reference genes (for qPCR conditions see^12,56,58^). Based on analysis with qbasePLUS software (Biogazelle, Zwijnaarde, Belgium^59^), *GAPDH*, *GLS*, *TBP* and *BACT* were chosen as optimal reference genes and were used for normalization. A multiple normalization factor was calculated for individual CL referring to Vandesompele *et al*.^60^.

### Western Blot analysis of SOD1 and SOD2

Protein homogenization and western blot analysis was done as described before^44^ with the following exceptions. Per 5 mg tissue 150 µl lysis buffer (assay buffer of Glutathione Peroxidase Assay Kit of abcam) were added. The protein concentrations of the lysates were determined by the method of Smith^61^. We applied 20 µg protein per SDS-PAGE lane. As primary antibodies mouse anti-SOD1 (1:2000, sc-101523, Santa Cruz Biotechnology, Inc., Heidelberg, Germany) and mouse anti-SOD2 (1:250, sc-137254, Santa Cruz Biotechnology) were used.

### In-gel activity assay of SOD2

In-gel activity assay for SOD2 was performed as described in Weydert & Cullen^62^. Briefly, CL homogenates (see 2.4) were applied onto native gels (20 µg protein per lane). After native PAGE run, the gel was incubated with SOD2 (MnSOD) staining solution in the dark for 20 min, thereafter it was washed twice with water before it was incubated in water under fluorescent light until the presence of SOD2 was visible by clear bands.

### Total SOD activity

For detection of SOD activity, a commercial kit (SOD determination kit, Sigma-Aldrich) and bovine SOD as standard were used. CL samples were homogenized as described before^44^, but using 150 µl sucrose buffer (0.25 M sucrose, 10 mM Tris, 1 mM EDTA, pH 7.4) per 1 mg tissue. Using the standard SOD with known activity in different dilutions we were able to generate a standard curve and calculating activity levels for our CL samples.

### Immunohistochemistry of SOD1 and SOD2

Immunohistochemistry was performed as described in^63^. The primary antibodies already listed under 4.4 were used in the following dilutions: anti-SOD1 1:100.000, anti-SOD2 1:100. As secondary antibody anti-mouse POD was used (ready to use solutions).

### Mass spectrometry

Mass spectrometry was performed on 8 CL samples, 4 fresh and 4 persistent ones. From IL1 and IL2 one fresh/persistent CL-pair was used per animal and from EL1 one pair of each ovary. Protein homogenates were prepared as described before^44^. From each sample, lysate containing 55 µg protein were applied on SDS-PAGE. After run, 10 bands per lane were excised from the gel, and in-gel tryptic digestion was performed as described before^64^. Peptides were analyzed by a reversed-phase capillary liquid chromatography system (Ultimate 3000 nanoLC system, Thermo Scientific) connected to an Orbitrap Elite mass spectrometer (Thermo Scientific) as described before^65^. The processed MS data were analyzed using the MaxQuant (1.5.9.3) software and searched in-house against the NCBInr_Felis_catus (Oct 2017) database (Oct 2017). Mass tolerance of precursor and sequence ions was set to 20 ppm and 0.35 Da, respectively. A maximum of two missed cleavages was allowed. Methionine oxidation and the acrylamide modification of cysteine were used as variable modifications. Peptides identifications were accepted if they based at least two identified (razor or unique) peptides. Quantifications were performed using label free quantification (LFQ) of the MaxQuant software (minimum ratio count 3).

### Statistical analysis

The statistical analysis of different stages was done as described before^65^. Briefly, cat samples were analyzed with the non-parametric Kruskal–Wallis rank sum test using the Wilcoxon rank sum test for post hoc pairwise comparisons (p-value adjustment: Benjamini–Hochberg). *Corpora albicantia* were excluded from this analysis. For the comparison of gene expression levels of fresh and persistent CL of individual lynxes, the Mann-Whitney U test was used. SigmaPlot (Systat Software Inc., San Jose, USA) was used to visualize the statistical results through box plots.

### Ethical statement

The study was approved by the Internal Committee for Ethics and Animal Welfare of the Leibniz Institute for Zoo and Wildlife Research in Berlin, Germany (Permit numbers: 2010-10-01 and 2011-01-01).

## Supplementary information

Supplementary informations.
