# Supplementary material for: The antioxidative enzyme SOD2 is important for physiological persistence of corpora lutea in lynxes
Source: Sci Rep. 2020 Feb 28;10:3681. doi: 10.1038/s41598-020-60634-x (PMC7048870; doi:10.1038/s41598-020-60634-x)
Supplement: Supplementary file 1 — Supplementary informations. [file 41598_2020_60634_MOESM1_ESM.pdf]

Supplemental Material

The antioxidative enzyme SOD2 is important for physiological persistence of *corpora lutea* in lynxes

B.C. Braun[1*], N. Halaski[1], J. Painer[2,3], E. Krause[4], K. Jewgenow[1]

[1] Leibniz Institute for Zoo and Wildlife Research, Department of Reproduction Biology, Alfred-Kowalke-Str. 17, 10315 Berlin, Germany

[2] Leibniz Institute for Zoo and Wildlife Research, Department of Reproduction Management, Alfred-Kowalke-Str. 17, 10315 Berlin, Germany

[3] present address: Veterinary University Vienna, Research Institute for Wildlife Ecology, Savoyenstreet 1, 1160 Vienna, Austria

[3] Leibniz-Forschungsinstitut für Molekulare Pharmakologie, Robert-Rössle-Str. 10, 13125 Berlin, Germany

[*] Corresponding author: braun@izw-berlin.de

Supplemental Table 1. Mass spectrometry analysis of lynx CL. For proteins analyzed in this study, numbers of peptides as well as the Label-free quantification values (LQF) intensities are listed. Samples: persistent (pe) and fresh (fr) CL of each Iberian lynx (IL1 and IL2) and of each ovary (-1, -2) of the same Eurasian lynx (EL1)

			**Razor + unique peptides**								**LFQ intensity**							
**analyzed in qPCR**	**Protein IDs**	**Peptide counts (unique)**	**IL1-fr**	**IL1-pe**	**IL2-fr**	**IL2-pe**	**EL1-1-fr**	**EL1-1-pe**	**EL1-2-fr**	**EL1-2-pe**	**IL1-fr**	**IL1-pe**	**IL2-fr**	**IL2-pe**	**EL1-1-fr**	**EL1-1-pe**	**EL1-2-fr**	**EL1-2-pe**
CAT	XP_003993206.1, PREDICTED: catalase [*Felis catus*]	25	20	22	22	22	23	15	22	20	1.994.800.000	2.133.100.000	891.410.000	636.150.000	579.450.000	366.840.000	687.000.000	1.496.800.000
GLRX3	XP_019669563.1, PREDICTED: glutaredoxin-3, partial [*Felis catus*]	7	4	4	6	6	3	2	4	2	22.576.000	11.936.000	26.706.000	20.675.000	18.919.000	0	8.705.000	0
GPX4	XP_011286531.2, PREDICTED: phospholipid hydroperoxide glutathioneperoxidase, mitochondrial, partial [*Felis catus*]	10	*4*	*8*	*8*	*6*	*4*	*3*	*1*	*8*	34.937.000	194.910.000	94.513.000	28.593.000	0	71.411.000	0	290.490.000
GSTP	XP_011285433.1, XP_019668324.1, PREDICTED: glutathioneS-transferaseP [*Felis catus*]	11;11	*5*	*8*	*6*	*8*	*5*	*4*	*5*	*8*	365.370.000	1.344.100.000	765.230.000	2.749.300.000	2.739.200.000	5.931.600.000	263.360.000	8.894.900.000
PRDX6	XP_011289183.1, PREDICTED: peroxiredoxin-6 [*Felis catus*]	25	14	17	21	23	20	16	15	18	826.850.000	1.037.500.000	420.630.000	719.000.000	1.350.500.000	1.462.100.000	632.520.000	1.378.000.000
PXDN	XP_019683554.1, XP_006930569.1, PREDICTED: peroxidasin homolog isoforms X1 and X2 [*Felis catus*]	9;9	3	3	9	7	5	2	5	1	9.605.900	8.713.300	107.440.000	20.014.000	22.840.000	0	15.984.000	0
SOD1	XP_006935984.1, PREDICTED:superoxidedismutase[Cu-Zn] [*Feliscatus*]	12	7	8	9	8	10	8	10	11	2.305.800.000	2.770.700.000	2.781.600.000	1.529.100.000	3.032.900.000	1.646.600.000	5.958.300.000	11.740.000.000
SOD2	XP_019687004.1, PREDICTED: superoxidedismutase[Mn] ,mitochondrial [*Felis catus*]	19	*8*	*14*	*13*	*17*	*11*	*12*	*10*	*16*	576.080.000	2.840.200.000	551.390.000	3.408.900.000	904.070.000	2.988.200.000	261.450.000	14.176.000.000
TXN	XP_011286804.1, PREDICTED: thioredoxin [*Felis catus*]	7	4	3	7	6	6	6	4	6	183.880.000	873.790.000	2.019.000.000	2.473.500.000	167.470.000	985.830.000	899.540.000	1.837.200.000
TXNRD2	XP_006938766.1, XP_019670460.1, XP_019670459.1, XP_019670458.1, XP_019670457.1, XP_019670456, XP_006938765.1, PREDICTED: thioredoxinreductase 2, mitochondrial isoforms X1 - X6 [*Felis catus*]	14;14;14;14;14;14;14	9	11	6	8	12	8	8	10	70.480.000	181.290.000	38.516.000	113.050.000	164.940.000	216.850.000	146.280.000	93.318.000

Supplemental Table 2. Samples numbers of CL used for qPCR and total-SOD-activity assay

Stages	qPCR	SOD-Assay
**domestic cat, non-pregnant stages (animals: n = 47)**		
formation	9	
development/maintenance	14	
early regression	14	
late regression	10	
**domestic cat, pregnant stages (animals: n = 22)**		
pre-implantation (day 2-10)	6	
post-implantation (day 14-36)	11	
regression (day 38-d49)	5	
**domestic cat, *corpus albicans* (animals: n = 4)**	4	
**Iberian lynx 1 (animal: n = 1, 7 days after mating)**		
fresh (formation)	3	2
persistent (development/maintenance)	8	1
**Iberian lynx 2 (animal: n = 1, 7 days after mating)**		
fresh (formation)	5	3
persistent (development/maintenance)	6	2
**Eurasian lynx 1 (animal: n = 1, ca. day 15-18 of pregnancy)**		
fresh (development/maintenance)	5	4
persistent (development/maintenance and/or early regression)	6	2
**Eurasian lynx group (animals: n = 5, non-breading season)**		
persistent (early regression)	18	6

Supplemental Figure 1

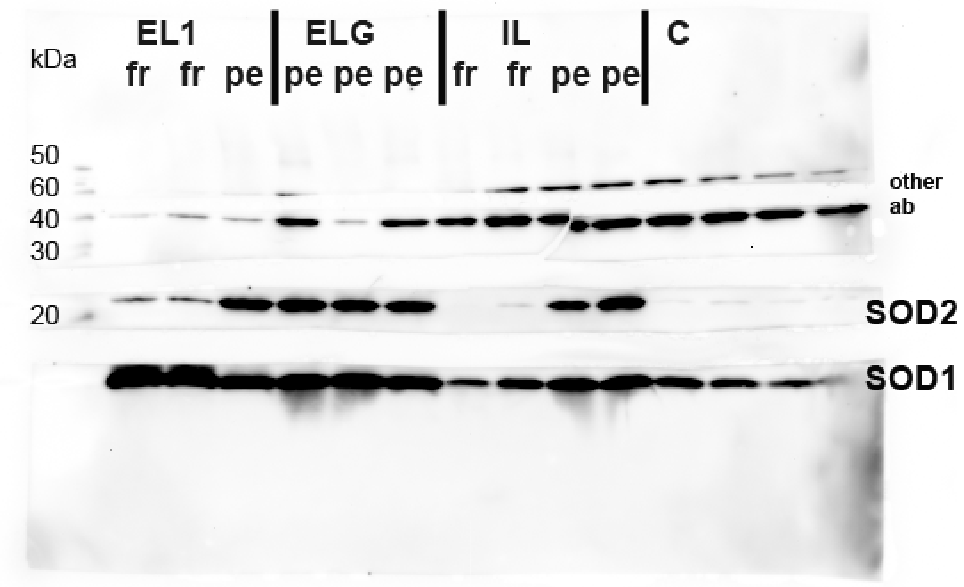

Supplemental Fig. 1: Western Blot for SOD1 and SOD2 detection - original blot (5 min exposure time) with labeling. Western blot membrane was divided after blotting and incubated with different primary antibodies. EL1: Eurasian lynx 1, ELG: samples of Eurasian lynx group, IL: samples of Iberian lynxes, C: samples of domestic cat CL, fr: fresh CL, pe: persistent CL, ab: antibodies

Supplemental Figure 2

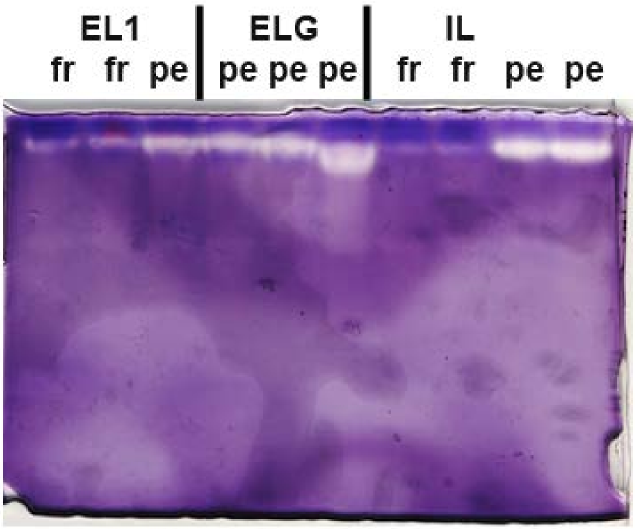

Supplemental Fig. 2: In-gel activity assay for SOD2 activity detection with CL homogenate samples of lynxes - original gel image with labeling. EL1: Eurasian lynx 1, ELG: samples of Eurasian lynx group, IL: samples of Iberian lynxes, fr: fresh CL, pe: persistent CL

Supplemental Figure 3

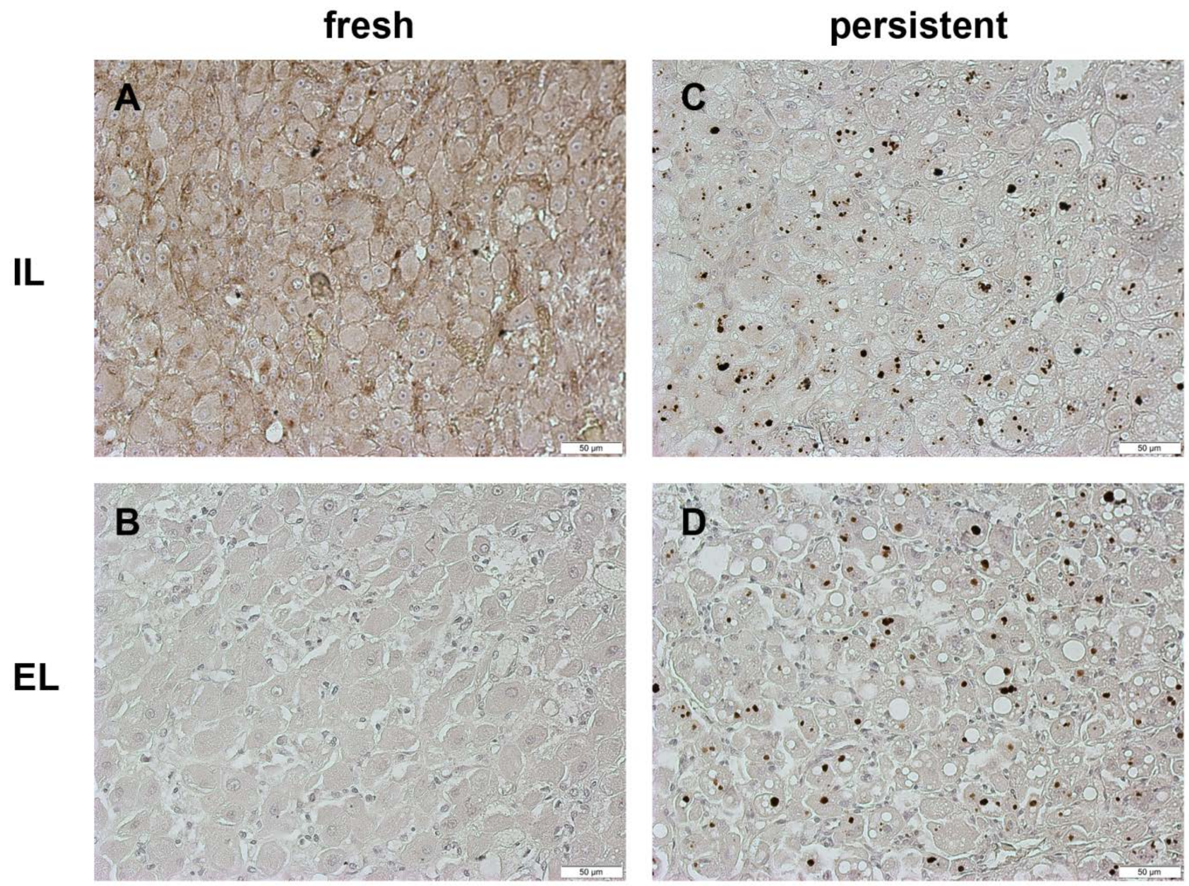

Supplemental Fig. 3: Immunohistochemical localization of NF-κB2 in lynx CL. Immunohistochemistry was performed on fresh (A, B) and persistent (C, D) CL samples of Iberian lynx (IL; A, C and Eurasian lynx (EL; B, D) with an anti-NFκB p52 antibody (Santa-Cruz sc-7386, dilution: 1:1000).
